# 

**Published:** 1887-01

**Authors:** 


### CONTENTS, JAN. 1887, (VOL. II, NO. VII.)

ORIGINAL ARTICLES—The Intimate Connection Between Areas of Low Barometer and the Prevalence of Epidemics of Non-Contagious Diseases, by T. C. Osborn, M. D., Cleburne, Texas........ .................... 264

A Case of Placental Presentation, by S. L. Lowry, A. M., M. D., San Antonio, Texas........................ 273

CORRESPONDENCE—Poisoning by Morphine Successfully Treated With Strychnia, by Dr. W. M. Barry, Abilene, Texas ....................................... 274

SOCIETY NOTES—The Central Texas Medical Association Meeting ....................................... 276

Action of the Galveston Medical Club, With Reference to the Appointment of State Health Officer.......... 278

East Texas Medical Association, 2nd regular meeting.... 279

From the Minutes of the Regular Annual Meeting of the West Texas Medical Association, Frederick Terrell, M. D., Secretary................................ 281

Physicians' Mutual Benefit Association.—Mrs. Welch's Receipt for Death Benefit Fund.................... 281

Errata: Dr. Wallace's Review of the Prize Essay...... 281

EDITORIAL—Some New(?) Uses for Carbolic Acid........ 282

Mineral Waters in Malarial Diseases.................. 284

Dr. Osborn's Paper in This Issue..................... 285

"Baking Powders" and the Public Health............. 286

BIBLIOGRAPHY—A Reference Hand Book of the Medical Sciences ....................................... 288

Hand Book of General Therapeutics................. 289

The Pharmacopœia of the United States of America.... 289

The Journal of Comparative Medicine and Surgery..... 291

Index Catalogue of the Surgeon-General's Office........ 292

A Manual of Dieteties............................. 292

TheHealing of Arteries............................ 292

Physicians' Visiting List for '87; Courier Revew Call Book; Physicians Hand Book..................... 293

Vicks' Floral Guide............................... 294

MEDICAL NEWS AND MISCELLANY—Twenty-five Items....294–299

Gaillard's Medical Journal.......................... 297

The New Administration and the State Health Officer.. 298

Appointments and Removals.........................298–299

ADVERTISER'S NOTICES—All interesting; read them, thirty in number; a pretty good showing..................300–303

